# A 10-week randomized controlled hip-strengthening intervention in healthy, well-trained runners: longitudinal and cross-sectional analyses of frontal-plane running biomechanics across running speeds

**DOI:** 10.1186/s13102-026-02148-z

**Published:** 2026-10-06

**Authors:** Sebastian Landauer, Ansgar Schwirtz, Florian Kurt Paternoster

**Affiliations:** Associate Professorship of Biomechanics in Sports, TUM School of Medicine and Health, Technical University of Munich, Am Olympiacampus 11, Munich, 80809 Germany

**Keywords:** Biomechanical phenomena, Hip, Muscle strength, Risk factors, Running

## Abstract

**Background:**

Hip abductor (HABDS) and hip external rotator strength (HERS) are proposed to influence frontal-plane running biomechanics, including characteristics associated with running-related injury risk. Evidence and intervention effects are inconsistent. Sex-specific differences in running biomechanics suggest that the relationship between hip strength and running biomechanics may differ between females and males. Whether relationships or the effects of hip strengthening are consistent across running speeds is unknown. This study aimed to examine the longitudinal effects of isolated hip strengthening on running biomechanics at three speeds and whether hip strength predicted running biomechanics at these speeds, accounting for sex.

**Methods:**

Forty-eight (23 females, 25 males) healthy, well-trained runners underwent baseline hip strength testing (motor-driven dynamometer) and 3D running analysis at three individually prescribed speeds based on 1000-m time: slow (60%), medium (80%), and fast (100%), corresponding to 2.76 ± 0.29, 3.69 ± 0.39, and 4.61 ± 0.49 m/s (mean ± SD). The primary outcome was frontal-plane knee moment, with frontal-plane hip, knee, and pelvis angles as secondary outcomes. Participants were randomized to a 10-week hip-strengthening intervention or a control group. Longitudinal changes were assessed using SPM t-tests of pre–post change scores between groups, conducted separately by sex and running speed. No sex × intervention or speed × intervention interactions were tested. Strength adaptations were evaluated using analysis of covariance. Cross-sectional analyses used SPM general linear models to determine whether hip strength predicted running biomechanics across running speeds, with sex as a covariate.

**Results:**

The intervention increased HABDS by 0.20–0.29 Nm/kg (10.9–16.7%), with significant effects for five of six conditions, and increased left eccentric HERS by 0.05 Nm/kg (5.9%). Biomechanical outcomes did not change. At baseline, greater HABDS was associated with higher knee abduction moments during late stance at slow running speed only. No other associations were significant.

**Conclusion:**

A 10-week hip abductor and external rotator strengthening program improved HABDS but did not alter frontal-plane running biomechanics in healthy, well-trained runners. Although greater HABDS was associated with higher knee abduction moments during slow running, the findings do not support a consistent relationship between hip strength and frontal-plane running biomechanics.

**Supplementary Information:**

The online version contains supplementary material available at https://doi.org/10.1186/s13102-026-02148-z .

## Introduction

Running is one of the most popular physical activities globally [1] and is consistently linked to substantial health benefits [2–4]. Despite these benefits, running is also accompanied by a considerable risk of musculoskeletal injury, with incidences and prevalences of ~ 40% and 45%, respectively [5]. Injury history is among the strongest non-modifiable risk factors for future injuries [6–8], whereas biomechanical factors offer a potentially modifiable pathway to risk reduction [9, 10].

Hip abductor and external rotator strength (HABDS/HERS) have been proposed as modifiable contributors to lower-extremity control during running. Deficits in HABDS/HERS have been associated with running-related injuries, including iliotibial band syndrome [11, 12]. Powers (2013) proposed that insufficient HABDS may impair control of pelvic motion during running and contribute to greater hip adduction [13]. These proximal alterations have been proposed to influence frontal-plane knee alignment and loading, while reduced HERS may additionally be associated with altered frontal- and transverse-plane lower-extremity mechanics [14]. Consistent with this proposed mechanism, increased hip adduction during running has been associated with increased risk of running-related injury [15–17]. Because the knee represents the most common site of running-related injuries [14, 18], understanding whether improvements in proximal hip function are associated with changes in knee joint loading is of particular clinical relevance.

The knee abduction moment is a measure of frontal-plane knee loading that integrates the combined effects of lower-limb kinematics, ground reaction forces, and muscle actions. Although knee abduction moments have been consistently linked to injury risk during landing and cutting tasks [19, 20], their role in the development of running-related injuries remains less well established. Nevertheless, evaluating the knee abduction moment provides an opportunity to determine whether changes in proximal hip function are accompanied by changes in distal knee loading during running, thereby addressing a clinically relevant consequence of hip-focused interventions.

The existing intervention literature further supports this rationale. Previous running-specific hip-strengthening studies have reported changes in knee abduction moments following training, whereas changes in frontal-plane hip kinematics have been less consistently observed [21, 22]. This suggests that biomechanical adaptations following hip strengthening may be reflected in knee loading even when changes in proximal kinematic variables are small or absent. Pelvis, hip, and knee frontal-plane kinematics, therefore, provide important mechanistic information for interpreting changes in knee loading and for improving our understanding of the biomechanical pathways through which hip strengthening may influence running mechanics.

However, causal inferences remain limited because most studies are cross-sectional or retrospective [23, 24]. It therefore remains unclear whether reduced hip strength contributes to injury development or is a consequence of it [11]. These temporal relationships require prospective research assessing hip strength and biomechanics before injury occurs.

The few available intervention studies have reported inconsistent effects of HABDS/HERS strengthening on running biomechanics [21, 22, 25, 26]. However, interpretation of these findings is limited by methodological factors, including the use of injured populations [21, 25], multimodal interventions combining strengthening with gait retraining or other exercises [26, 27], and the absence of appropriate control groups [21, 22]. Therefore, the isolated effects of HABDS/HERS strengthening on running biomechanics remain unclear and require investigation in well-controlled trials involving healthy runners.

Furthermore, hip strength may influence running mechanics primarily under more demanding conditions, when frontal- and transverse-plane loading demands are increased. Higher running speeds or fatigue may amplify the contribution of the hip musculature to maintaining lower-limb alignment [24, 28]. However, it remains unclear whether strength-biomechanics relationships are evident across different running speeds.

Another factor that may influence the relationship between hip strength and running biomechanics is sex. Female runners have a higher incidence of running-related injuries than male runners [18] and exhibit distinct biomechanical characteristics, including greater hip adduction and hip internal rotation during running [29]. Given that hip strength may influence lower-extremity biomechanics, sex may therefore be an important consideration when investigating strength-biomechanics relationships.

Overall, cross-sectional associations between HABDS/HERS and running biomechanics remain inconsistent across joints and planes and may depend on speed. Intervention studies are few, often involve injured participants, and frequently combine strengthening with other modalities. Well-controlled trials in healthy runners that isolate the effects of HABDS/HERS on running biomechanics are therefore needed.

Hence, this study primarily aimed to determine whether a 10-week isolated HABDS/HERS strengthening program is associated with changes in running biomechanics compared with a control group, with intervention analyses conducted separately by sex and running speed. Secondarily, it aimed to examine whether baseline HABDS/HERS predicts running biomechanics across three running speeds, with sex included as a covariate, providing complementary information on the cross-sectional relationship between hip strength and running mechanics. It was hypothesized that the intervention would lead to reductions in knee abduction moments (primary outcome) and in pelvic drop, hip adduction, and knee abduction angles (secondary outcomes). For the cross-sectional analyses, it was hypothesized that greater HABDS/HERS would predict lower baseline values of these biomechanical variables. Running speed was examined separately in the intervention and cross-sectional analyses to characterize the consistency of findings across speeds; differences between speeds were interpreted descriptively because no formal speed × intervention or speed × strength interaction tests were performed. No formal sex × intervention interaction test was performed.

## Methods

### Participants

An a priori power analysis (GPower 3.1.9.7) for a repeated-measures design (with intervention, sex, and time as factors) was conducted assuming a medium effect size (f = 0.25) according to Cohen’s conventions [30], as previous hip-strengthening intervention studies reported heterogeneous biomechanical outcomes that did not allow derivation of a reliable expected effect size for this design. With α = 0.05, power = 0.80, two time points, and a correlation of 0.5 between repeated measures, the required sample size was estimated at 48 participants. Because the final SPM1D analyses did not permit the planned repeated-measures model, the power calculation was based on the originally planned design and should therefore be regarded as an approximation rather than a calculation based on the exact final statistical model. Because the planned waveform analyses were performed using SPM1D, which does not support repeated-measures models with multiple factors and covariates, intervention effects were assessed using pre-post change scores. For a two-group, two-time-point design, an independent comparison of change scores tests whether the magnitude of change differs between groups and therefore addresses the same group × time question as the corresponding two-time-point repeated-measures analysis. This approach was used for the SPM1D intervention analyses, which were conducted separately by sex and running speed. Allowing for a 20% dropout rate, 56 participants were recruited from local running clubs and the university via email and flyers. Eight participants withdrew due to illness or scheduling conflicts, resulting in a final sample of 48 (Table 1). Participants were block-randomized to intervention or control using a custom MATLAB script, version R2025a (The MathWorks, Inc., Natick, MA, USA).

**Table 1:** Baseline demographic and running-related characteristics of participants in the control, intervention, and total sample

Characteristic	Control (*n* = 22)	Intervention (*n* = 26)	Total (*n* = 48)
Age (years)	27.1 ± 4.1	32.3 ± 9.7	29.9 ± 8.0
Height (m)	1.75 ± 0.09	1.74 ± 0.07	1.74 ± 0.08
Mass (kg)	69.8 ± 11.8	69.0 ± 8.6	69.4 ± 10.1
Body mass index (kg/m^2^)	22.8 ± 2.4	22.7 ± 2.0	22.7 ± 2.2
Running experience (years)	8.3 ± 6.4	6.4 ± 3.8	7.3 ± 5.2
Weekly mileage (km/week)	33.2 ± 21.6	29.2 ± 22.7	31.1 ± 22.1
1000 m time (s)	216.7 ± 27.7	221.6 ± 17.7	219.4 ± 22.7
Sex (female/male)	12/10	11/15	23/25

Continuous variables are presented as mean ± standard deviation. Sex distribution is shown as female/male counts

Inclusion criteria were age ≥ 18 years, > 2 years of regular running (≥ 2 sessions/week), and a 1000 m time < 4:30 min (females) or < 4:00 min (males), determined from an all-out 1000 m run performed during the baseline laboratory session. These sex-specific thresholds were selected pragmatically to ensure a sample of trained recreational runners with sufficient running experience and ability. Exclusion criteria were lower-limb injury or surgery within 12 months, current running-related pain, or any unresolved “YES” on the PAR-Q+, a pre-participation screening tool [31]. All participants provided written informed consent, and the study protocol was approved by the University Ethics Committee (2024-491-S-CB / 2024) and conducted in accordance with the Declaration of Helsinki.

### Data collection procedure

Participants completed three laboratory sessions at the Technical University of Munich. First, they attended a baseline session including strength testing (HABDS/HERS) and a 1000 m all-out run. Second, at least three days later, they completed a running biomechanics assessment, were allocated to the intervention or control group, and participated in the first supervised intervention session. Third, after the intervention (or control) period, they returned for post-intervention testing, including running biomechanics and strength assessments. Assessors were blinded except during post-intervention running analysis, as the same researcher who instructed participants during the supervised intervention session also conducted this assessment. This researcher therefore had access to group allocation during post-intervention data processing. Biomechanical processing was performed using pre-specified MATLAB scripts developed prior to the main analysis and tested using pilot data, thereby minimizing subjective processing decisions.

#### Strength testing

**Fig. 1:**
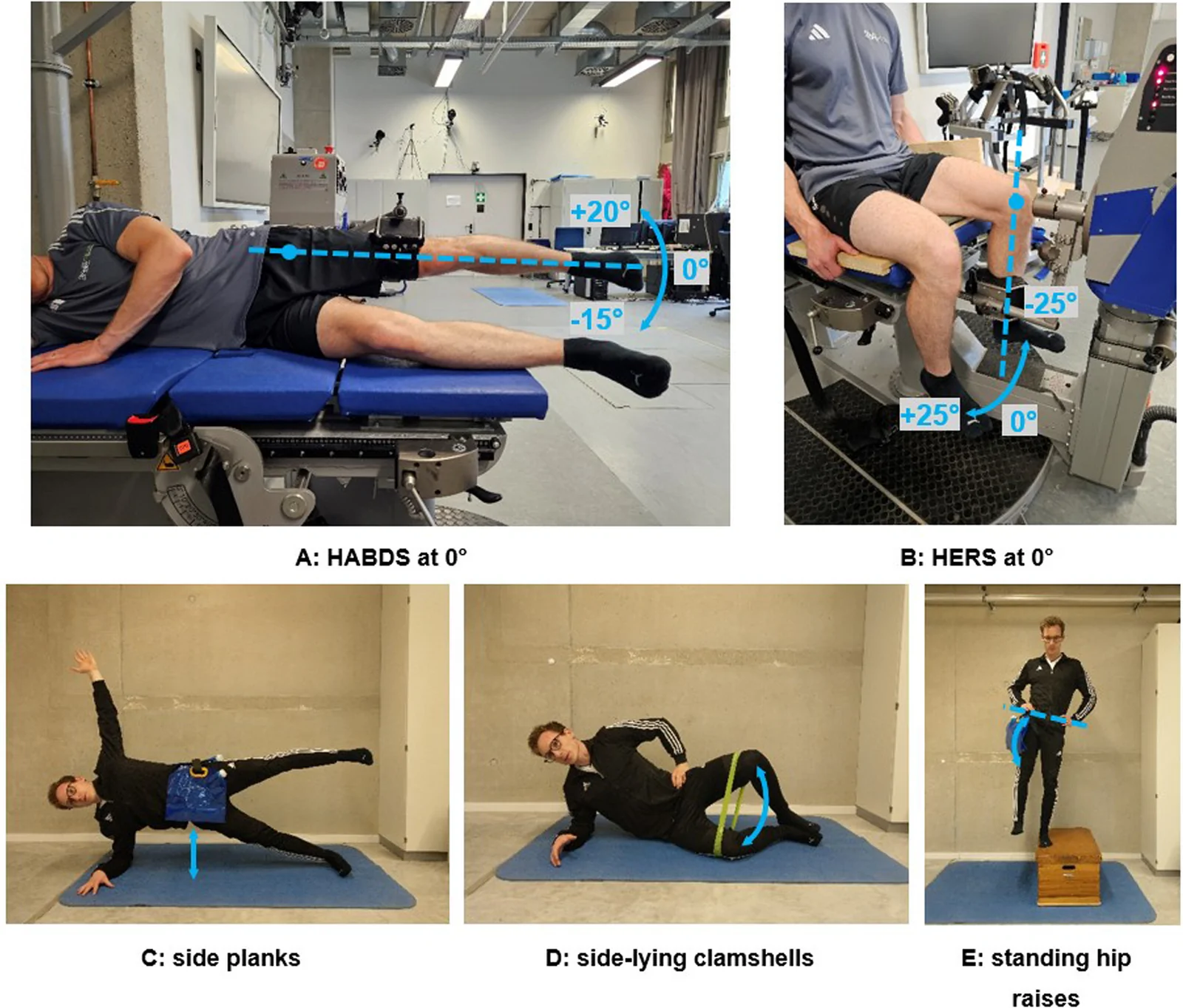
Strength testing setup and intervention exercises. Strength testing at 0° (neutral posture) for hip abductor strength (HABDS, **A**) and hip external rotator strength (HERS, **B**). Intervention exercises include side planks (**C**), side-lying clamshells (**D**), and standing hip raises (**E**)

Strength was measured using a motor-driven dynamometer, Isomed 2000 (D. & R. Ferstl GmbH, Hemau, Germany), capturing torque and angles at 1000 Hz. Hip abductor and external rotator strength were assessed under isometric, concentric, and eccentric conditions to comprehensively characterize the strength adaptations induced by the intervention. However, only isometric strength was included in the primary biomechanical analyses. Isometric strength was selected a priori as the primary predictor because it is the most established strength measure in the existing literature on hip strength and running biomechanics. In contrast, concentric and eccentric outcomes were evaluated as secondary strength outcomes to provide a complete profile of the intervention-induced adaptations. Participants were side-lying for HABDS (Fig. 1A) and seated for HERS (Fig. 1B). For HABDS, the lever arm was secured to the resistance pad positioned against the lateral thigh, 2 cm proximal to the patella. For HERS, it was secured to the resistance pad aligned with its lower edge at the medial malleolus. The dynamometer axis was aligned with the hip joint center for both tests. Similar hip abductor and external rotator strength testing positions have previously been used in healthy participants, with hip abductor strength assessed in a side-lying position and hip external rotator strength assessed in a seated position [22]. The reliability of strength measurements using the ISOMED 2000 has also been demonstrated across several muscle groups and testing modes [32].

Neutral position (0°) corresponded to a natural upright standing posture for HABDS and vertical knee-ankle alignment for HERS, and served as the reference position for the isometric strength assessments. Dynamic trials (concentric and eccentric) were performed through a predefined range of motion (RoM) from − 15° adduction to + 20° abduction (HABDS) and from − 25° internal to + 25° external rotation (HERS) at 30°/s. These RoMs were determined during pilot testing to allow participants to achieve the prescribed angular velocity (30°/s) at the neutral position during dynamic contractions.

After brief familiarization with the testing procedure immediately before testing and 10 submaximal repetitions, gravity correction was performed, followed by one maximum voluntary isometric contraction (MVC) at the start position (HABDS: −15°/+20° adduction–abduction; HERS: −25°/+25° internal–external rotation for concentric/eccentric testing). This MVC was used to determine the 95% MVC threshold for initiating dynamic trials. Participants then performed 3 MVCs per side per mode. Isometric trials lasted 3 s at neutral.

Concentric and eccentric trials were triggered once participants reached 95% of their isometric MVC. The trigger ensured a high and consistent level of muscle activation before movement onset and promoted fascicle lengthening during eccentric trials, consistent with previous isokinetic testing protocols [33]. Participants then exerted maximal effort throughout the full RoM. Standardized verbal encouragement and 60–90 s rest were provided. Exercise order, side, and contraction mode were block-randomized.

The selected testing positions were based on previous studies investigating hip strength and running biomechanics. Snyder et al. assessed hip abductor strength in a side-lying position and hip external rotator strength in a seated position, corresponding to the positions used in the present study [22]. However, a handheld dynamometer secured with straps was used instead of a stationary one [22]. Previous studies have also reported good-to-excellent reliability of strength measurements using the ISOMED 2000 across different muscle groups and testing modes [32, 34].

#### Running analysis

Participants wore their own shoes, identical at pre- and post-testing. They ran on an 18-m runway marked every 6 m and paced by acoustic cues. Individual target speeds were based on prior 1000-m time: fast (100%), medium (80%), and slow (60%), with 100% corresponding to each participant’s average velocity during the 1000-m performance test, resulting in 4.61 ± 0.49, 3.69 ± 0.39, and 2.76 ± 0.29 m/s (mean ± standard deviation). Speed was monitored with photocells (Microgate Witty-Gate, Bolzano, Italy) at 12–18 m. Trials within ± 5% of the individual target speed were accepted. The ± 5% threshold was selected based on pilot testing to ensure sufficient separation among the three target speed levels while remaining practically achievable. The percentage deviation from the target speed was assessed immediately after each trial and only trials within ± 5% of the target speed were included. Five trials per leg and speed were recorded (2 × 5 × 3). Speed blocks were randomized.

Ground reaction forces were sampled at 1000 Hz using an embedded 90 × 60 cm 3D force plate, model BP900600 (Advanced Mechanical Technology, Inc., Watertown, MA, USA), while 3D kinematics were recorded at 250 Hz with a 15-camera motion capture system, 7 Vicon Vantage, 6 Vicon T-series, and 2 Vicon Bonita cameras (Oxford Metrics Ltd, Oxford, UK). Reflective markers were placed according to the lower-body Plug-in Gait model, with additional medial femoral epicondyle and medial malleolus markers, and thigh/tibia clusters. Medial knee and ankle markers were included in static calibration and dynamic trials to compute tibial and thigh axes from medial and lateral landmarks, ensuring alignment with the Plug-in Gait model and improving knee frontal-plane accuracy by minimizing model assumptions.

### Data processing

#### Strength data

Strength data were low-pass filtered with a zero-phase 2nd-order Butterworth filter at 3 Hz. The 3-Hz cutoff frequency was determined a priori based on pilot testing to reduce high-frequency noise while preserving the characteristics of the torque signal relevant to peak torque assessment. The same filtering procedure was applied across all participants and contraction conditions. For each contraction condition, peak torques were extracted at the neutral position and corrected for gravity. The highest value across 3 trials per limb and contraction mode was retained for analysis.

#### Running data

Marker trajectories were processed and joint kinematics and kinetics were calculated in Vicon Nexus, version 2.16 (Oxford Metrics Ltd, Oxford, UK), using the Plug-in Gait lower-body model. Automated gap filling (Woltring spline ≤ 5 frames and rigid-body gap filling ≤ 250 frames) and Woltring filtering (smoothing parameter: 15) were applied. Further processing was performed in MATLAB, and stance phases were extracted using initial contact and toe-off events defined by a 5 N vertical force threshold. Joint angles were calculated using the Cardan rotation sequence implemented in Vicon Nexus (YXZ sequence). Positive values represented ipsilateral pelvic elevation, hip adduction, knee adduction, and external knee abduction moments, with opposite signs representing the corresponding opposite directions. Joint angles and moments were low-pass filtered with a zero-phase 2nd-order Butterworth filter at 12 Hz. The 12-Hz cutoff frequency was determined a priori and applied consistently across all participants and running conditions, consistent with previously recommended cutoff frequencies [35–37]. The filtered data were then time-normalized to 101 points via linear interpolation. Trials were averaged per limb and speed for analysis. Joint kinematics and kinetics were evaluated for the predefined variables during the stance phase.

### Hip abductor and external rotator strengthening program

The 10-week HABDS and HERS training included one supervised session, followed by independent training 3x/week (20 min/session) using instructional videos and an exercise log. Self-reported session adherence was calculated as the number of completed sessions divided by the number of prescribed sessions and was 84 ± 12% (range: 60–110%). Among participants who completed the study, no participants were excluded on the basis of intervention adherence. Each session comprised 6 sets per limb across 3 exercises (Fig. 1C-E) in order: side planks, side-lying clamshells, and standing hip raises, with 90s rest between sets, ideally on non-consecutive days. Load was progressively adjusted to achieve 8–12 repetitions during weeks 1–6 and 6–8 repetitions during weeks 7–10, with 2 repetitions in reserve. This progression was intended to provide a sufficient, progressively increasing training stimulus while allowing resistance to be adjusted to each participant’s strength level. The reduced number of repetitions during the last 4 weeks aimed at maximal strength development using higher loads, consistent with the primary outcome measure. Movement velocity was standardized (< 1s concentric, >2s eccentric).

Side planks (Fig. 1C) were performed side-lying with hips lifted, supported on the forearm and knee or foot, with optional hip load. Clamshells (Fig. 1D) were side-lying with 90° knee flexion, lifting the top knee with loop-band resistance while maintaining pelvic stability. Standing hip raises (Fig. 1E) were performed unilaterally from a block for full RoM with optional pelvis load.

Resistance was adjusted individually whenever participants exceeded the prescribed repetition range while maintaining two repetitions in reserve. Side planks were initially performed from the knees or the feet, depending on the participant’s strength level. Progression of side planks occurred by advancing from the knee-supported to the foot-supported variation when participants were able to complete the prescribed repetition range. Further progression was achieved by increasing external load using water-filled containers. Standing hip raise loads were also increased using water-filled containers, whereas clamshell resistance was progressed using loop bands of increasing stiffness or, when required, combinations of multiple bands. Participants were provided with sufficient loading equipment to maintain the target repetition range throughout the intervention.

The exercise selection was designed to target the hip abductors and external rotators while allowing participants to complete the program independently at home with minimal equipment. Side planks and resistance-band clamshells were based on exercises used by Willy and Davis [26], who reported increases of 41% in HABDS and 24% in HERS following their intervention. The standing hip raises were adapted from the hip-raise exercise used by Snyder et al. [22], who reported increases of 12% in hip abductor strength and 23% in hip external rotator strength on the trained side. In Snyder et al. (2009), resistance was provided by a cable attached to a waist strap [22]. In the present study, water-filled containers were used to provide resistance during the same exercise, allowing home-based training.

The control group was instructed not to initiate any additional hip abductor or hip external rotator strengthening exercises during the 10-week intervention period, but was allowed to maintain their habitual strength training activities. Both groups were instructed to maintain their habitual running volume and intensity throughout the 10-week intervention period. Weekly running volume was monitored using running watches and maintained within ± 10% of baseline.

### Statistical analysis

Statistical parametric mapping (SPM) for joint angles and moment waveform data was performed in MATLAB using SPM1D (v0.4.12; www.spm1d.org), based on the framework described by Pataky [38].

For the intervention analyses, between-group differences in change scores (pre-post) were assessed using independent SPM t-tests, conducted separately for each sex and running speed. This approach enabled evaluation of intervention-related changes across the complete biomechanical waveform within the analytical framework supported by SPM1D (see 4.3 Limitations).

For cross-sectional analyses, SPM general linear models (GLM) tested whether HABDS and HERS predicted running biomechanics. Models were fitted separately for each running speed, with strength as the predictor and sex included as a covariate (Y ~ strength + sex). Right isometric strength was defined a priori as the primary predictor for cross-sectional analyses, with the right limb selected to limit analytical complexity and the number of multiple comparisons. Right isometric strength was evaluated with Holm-Bonferroni correction for multiple comparisons. Biomechanical variables were extracted from the right limb during the stance phase of the right limb to match the primary strength predictor. No between-limb comparisons were performed. For visualization, scatter plots were generated at the representative cluster peak (i.e., the time point of maximal association within each significant cluster). Fitted regression lines derived from the GLM coefficients were plotted as well. These visualizations were generated solely to illustrate the statistically tested relationships identified by the SPM analyses.

For both GLM and t-tests, the primary outcome was the frontal-plane right knee moment and the secondary outcomes were the frontal-plane right hip, knee, and pelvis angles. Results are reported as cluster-level probabilities (p), cluster timing (% stance), and peak effect sizes (r_max_ for GLM, interpreted as small/medium/large at 0.10/0.30/0.50; and d_max_ for t-tests, interpreted at 0.20/0.50/0.80, respectively) [30, 39].

Between-group strength outcomes were additionally analyzed using ANCOVA, with post-intervention strength values as the dependent variable and baseline strength and sex included as covariates. Sex was included to account for potential sex-related differences in strength outcomes. ANCOVA was selected because baseline-adjusted comparisons are recommended for randomized intervention studies with scalar outcomes [40]. Analyses were conducted separately for each exercise, tested limb, and contraction mode to describe strength adaptations following training of both limbs. No between-limb comparisons were performed. Partial eta squared (η^2^_p_, interpreted as small/medium/large at 0.01/0.06/0.14, respectively) [30, 41] was reported as the effect size. Mean within-group percentage changes with 95% confidence intervals are presented descriptively.

The Holm-Bonferroni correction (p_Holm_) controlled the familywise error rate for confirmatory SPM analyses. For the intervention t-tests, the primary outcome comprised one waveform variable tested separately for females and males and across three running speeds, resulting in 6 SPM waveform tests (1 × 2 × 3). The three secondary waveform variables were likewise tested separately for females and males and across three running speeds, resulting in 18 SPM waveform tests (3 × 2 × 3). For the cross-sectional GLM analyses, sex was included as a covariate rather than as separate groups. Thus, the primary outcome comprised 3 SPM waveform tests across the three running speeds (1 × 3), and the three secondary outcomes comprised 9 SPM waveform tests (3 × 3). Holm-Bonferroni correction was applied separately to each of these four families of SPM tests (t-tests: primary *n* = 6, secondary *n* = 18; GLM: primary *n* = 3, secondary *n* = 9). Adjusted p-values were computed using the Holm-Bonferroni step-down procedure and are reported as monotonic, such that each adjusted p-value is at least as large as the preceding one. Statistical significance was set at α = 0.05 and all SPM tests were two-tailed.

## Results

### Intervention

#### Primary and secondary variables

No significant between-group differences in pre-post waveform changes were observed for frontal-plane knee moments (primary) or frontal-plane pelvis, hip, and knee angles (secondary) after Holm-Bonferroni correction across sexes and running speeds (Table 2). Baseline biomechanical waveforms for the intervention and control groups are shown descriptively in Supplementary Figs. 1–4.

**Table 2:**
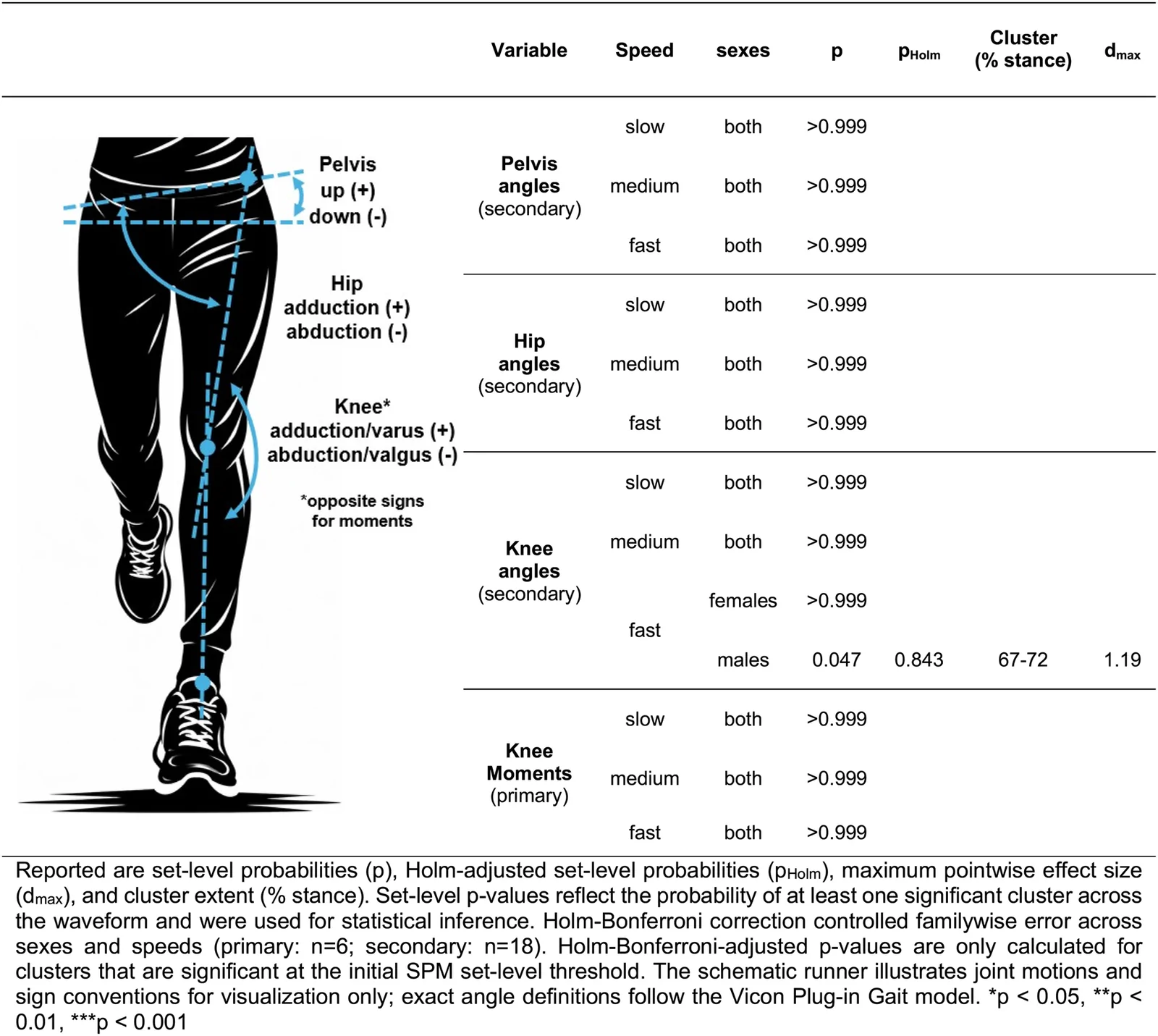
SPM two-sample t-test results comparing pre-post waveform changes between intervention and control groups, for frontal-plane right knee moment (primary) and frontal-plane right hip, knee, and pelvis angles (secondary) across sexes, limbs, and running speeds

#### Strength adaptations

ANCOVA adjusted for baseline values and sex (Table 3) revealed significant group effects for HABDS across contraction modes and sides after Holm-Bonferroni correction. Left-limb effects were significant for concentric (t(48) = 3.12, p_Holm_=0.029, η^2^_p_ = 0.18) and eccentric strength (t(48) = 3.10, p_Holm_=0.029, η^2^_p_ = 0.18). Right-limb HABDS increased significantly for isometric (t(48) = 3.63, p_Holm_=0.007, η^2^_p_ = 0.23), concentric (t(48) = 4.32, p_Holm_=0.001, η^2^_p_ = 0.30), and eccentric contractions (t(48) = 3.73, p_Holm_=0.006, η^2^_p_ = 0.24). Descriptively, mean within-group changes in the control group ranged from − 0.05 to + 0.05 Nm/kg (-2.4 to + 3.5%), whereas mean changes in the intervention group ranged from + 0.20 to + 0.29 Nm/kg (+ 10.9 to + 16.7%) across contraction modes and sides. These percentage changes are presented descriptively and do not represent statistical evidence of training effects (Supplementary Table 1).

**Table 3:** ANCOVA comparisons of hip abductor strength (HABDS) and hip external rotator strength (HERS) for left and right sides across contraction modes, with post-intervention strength as the dependent variable and baseline strength and sex included as covariates

Exercise	Side	Mode	*N*	Adjusted between-group difference, Nm/kg (95% CI)		t-value	η^2^_*p*_	*p*_Holm_
HABDS	Left	isometric	48		+ 0.17 (0.04 to 0.30)	2.66	0.14	0.054
		concentric	48		+ 0.18 (0.06 to 0.30)	3.12	0.18	0.029*
		eccentric	48		+ 0.24 (0.09 to 0.40)	3.1	0.18	0.029*
	Right	isometric	48		+ 0.23 (0.10 to 0.35)	3.63	0.23	0.007**
		concentric	48		+ 0.25 (0.14 to 0.37)	4.32	0.3	0.001**
		eccentric	48		+ 0.30 (0.14 to 0.46)	3.73	0.24	0.006**
HERS	Left	isometric	47		+ 0.05 (0.01 to 0.10)	2.39	0.12	0.085
		concentric	48		+ 0.04 (0.00 to 0.08)	2.11	0.09	0.122
		eccentric	48		+ 0.08 (0.03 to 0.13)	3.04	0.17	0.029*
	Right	isometric	48		+ 0.02 (-0.03 to 0.07)	0.78	0.01	0.440
		concentric	48		+ 0.03 (-0.01 to 0.07)	1.43	0.04	0.321
		eccentric	47		+ 0.07 (0.02 to 0.12)	2.77	0.15	0.050

Reported are adjusted between-group differences from the ANCOVA model (Post ~ Group + Pre + Sex), with 95% confidence intervals, t-values for the group effect, and partial eta squared (η^2^_p_) as effect size. Holm-adjusted p-values control the familywise error rate across all variables (12 tests). Full descriptive statistics, including pre- and post-intervention means, standard deviations, and absolute changes with 95% confidence intervals, are provided in Supplementary Table 1

*p < 0.05, **p < 0.01, ***p < 0.001

For HERS, ANCOVA (Table 3) showed a significant Holm-corrected effect only for left eccentric strength (t(48) = 3.04, p_Holm_=0.029, η^2^_p_ = 0.17). Descriptively, mean within-group changes in the control group ranged from − 0.04 to 0.00 Nm/kg (-4.5 to -0.2%), whereas mean changes in the intervention group ranged from + 0.01 to + 0.05 Nm/kg (+ 2.4 to + 5.9%). These percentage changes are descriptive and should not be interpreted as evidence of training effects. Confidence intervals generally overlapped zero except for left isometric and bilateral eccentric strength (Supplementary Table 1).

The distributions of individual changes are shown in Supplementary Fig. 5, illustrating within-group variability and group differences across contraction modes and limbs.

### Cross-sectional

**Fig. 2:**
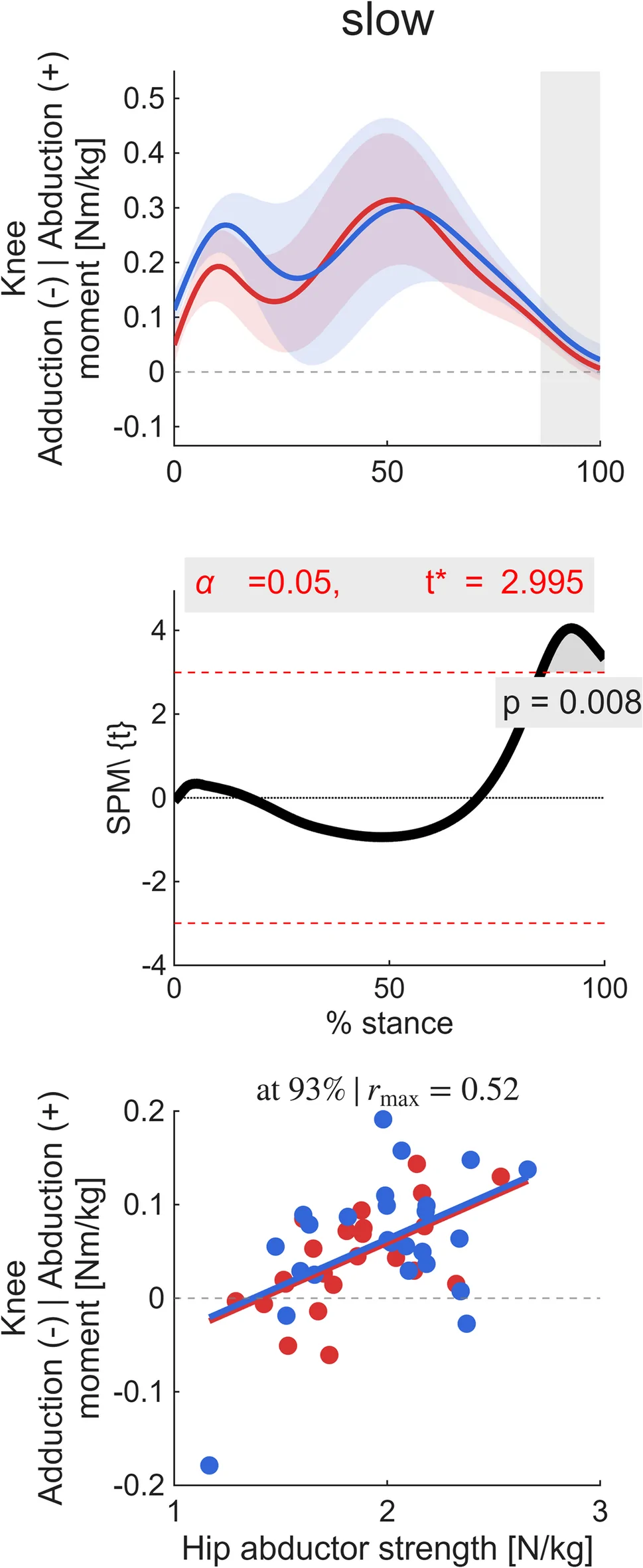
SPM GLM results showing the association between right isometric hip abductor strength (HABDS) and frontal-plane right knee moments during slow running, the only association that remained significant after Holm-Bonferroni correction across running speeds (p_Holm_=0.024). Top row: Mean bodyweight-normalized knee moments (Nm/kg) during stance for females (red) and males (blue), with 95% confidence intervals. Grey shading indicates the significant time interval. Middle row: SPM{t} from the sex-adjusted GLM (Y ~ strength + sex). The red dashed line denotes the critical threshold (α = 0.05); the significant cluster is annotated with the uncorrected set-level p-value. Bottom row: Scatter plot at the representative cluster peak (% stance) with fitted lines from the GLM (parallel slopes for females and males). This plot is descriptive. Statistical inference is based on SPM

After adjustment for sex, only one association remained significant after Holm-Bonferroni correction (Table 4). Specifically, greater HABDS was associated with higher knee abduction moments during late stance (86–100% stance; r_max_=0.52, p_Holm_=0.024) (Fig. 2). Several associations with hip and knee angles reached the uncorrected significance threshold (*p* < 0.05), but none remained significant after Holm-Bonferroni correction (all p_Holm_=0.364).

No significant associations were identified between HERS and the primary or secondary outcomes.

**Table 4:**
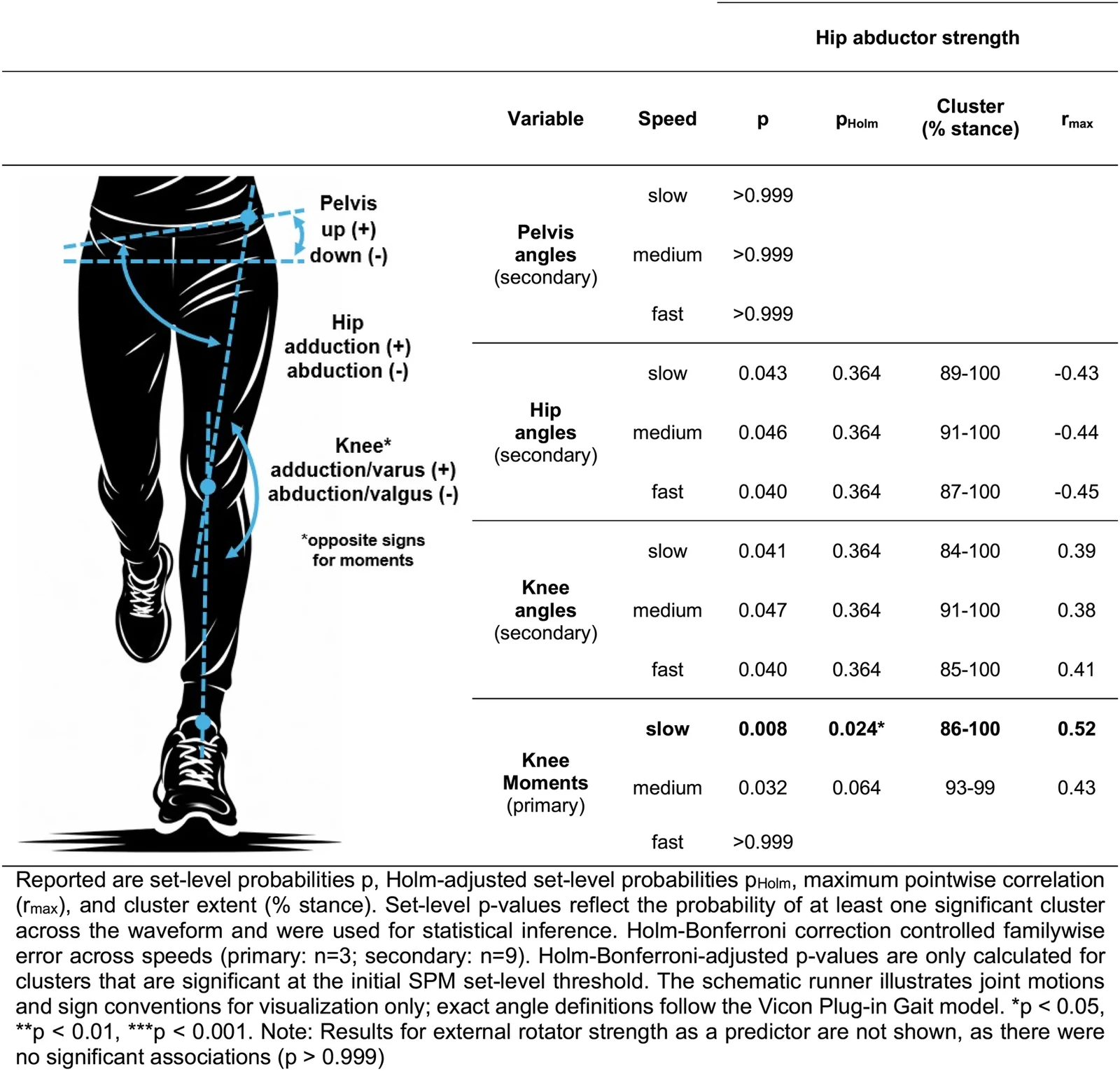
SPM GLM results examining right isometric hip abductor (HABDS) as predictors of frontal-plane right knee moments (primary) and frontal-plane hip, knee, and pelvis angles (secondary) across running speeds

## Discussion

This study primarily examined whether a 10-week isolated HABDS/HERS strengthening intervention reduces knee abduction moments (primary outcome) and pelvic drop, hip adduction, and knee abduction angles (secondary outcomes). Secondarily, it assessed whether greater HABDS/HERS predicts lower baseline values for these outcomes. For both analyses, outcomes were examined across three running speeds to explore whether strength–biomechanics relationships differed under varying running conditions.

### Main findings

Overall, 10 weeks of isolated strengthening of the hip abductors and hip external rotators did not produce consistent changes (Table 2) in running biomechanics in this cohort of healthy, well-trained runners. The relatively small sample and high training level of the participants should be considered when interpreting these null biomechanical findings, as the results may not generalize to less-trained or symptomatic runners.

This raises the question of whether the achieved strength gains were sufficient to induce measurable biomechanical adaptations. The intervention program (Table 3) was successful in increasing HABDS by + 0.20 to + 0.29 Nm/kg (10.9–16.7%) across contraction modes, whereas HERS improvements were modest with only + 0.01 to + 0.05 Nm/kg (2.4–5.9%) and limited to eccentric strength, falling short of previously reported adaptations (16–40%) [21, 22, 26]. Accordingly, the limited HERS adaptation may explain the absence of HERS-related biomechanical effects. However, the present study does not establish that a greater increase in strength would have produced such effects. Indeed, one recent study reported that strength increases of approximately 25–50% were associated with small changes in hip rotation during running [42]. This magnitude should not be interpreted as a threshold for biomechanical adaptation, as it is unknown whether a similar relationship applies to HERS and the biomechanical outcomes examined here.

Although this finding may indicate that the modest HERS gains contributed to the absence of HERS-related biomechanical effects, the same explanation is less convincing for HABDS. Even substantially larger HABDS improvements reported in previous studies (33–42%) have not consistently translated into meaningful changes in running mechanics [25, 26]. In contrast, more modest gains (10–12%), similar to those observed in this study, have been associated with reductions in variables such as knee abduction moments following targeted strength training [21, 22]. Notably, these effects have been observed in more specific populations, including runners with patellofemoral pain and physically active individuals who are not specialized in running. In contrast, the present study involved a mixed-sex sample of healthy, well-trained runners. Thus, the absence of biomechanical changes in the present study should be interpreted within the context of this relatively small, highly trained, asymptomatic sample and should not be readily generalized to populations with lower training status or existing running-related symptoms. Taken together, these findings indicate that biomechanical adaptations may depend more on context than on the magnitude of strength gains. Thus, despite the intervention successfully improving strength, insufficient adaptation is unlikely to fully explain the lack of consistent biomechanical changes, particularly for HABDS.

Although the intervention did not produce meaningful changes in running biomechanics, the cross-sectional analyses provided an opportunity to examine whether HABDS/HERS still predicted frontal-plane biomechanics at baseline. However, only one association remained significant after Holm–Bonferroni correction, suggesting that the present data do not support strong and consistent relationships between hip strength and frontal-plane running biomechanics.

Contrary to the primary hypothesis, greater HABDS was associated with higher rather than lower knee abduction moments during late stance at slow running speed (Table 4). This association was not observed at medium or fast running speeds. Given the limited evidence linking HABDS to knee frontal-plane loading in runners [27, 28, 43], the present finding does not support the assumption that greater HABDS is necessarily associated with reduced knee abduction moments in healthy runners. Importantly, this was a cross-sectional association and therefore does not indicate that greater HABDS causes higher knee abduction moments or that changes in HABDS would lead to changes in knee loading. Instead, these findings suggest that in this cohort of healthy, well-trained runners, greater hip abductor strength was not associated with reduced knee abduction moments during late stance at slow running speed, highlighting that strength adaptations may not directly translate into reductions in distal joint loading.

Beyond the association between HABDS and knee abduction moments, there was no other significant association after Holm–Bonferroni correction. HABDS was not associated with pelvic drop, consistent with previous studies [44–47], suggesting that pelvic stability during running is not determined solely by isolated hip strength. Associations with hip adduction and knee frontal-plane angles did not remain significant after correction either. Although the observed directions were consistent with previous reports [28, 45, 48–52] these patterns should be interpreted cautiously and do not provide evidence for consistent relationships between HABDS and frontal-plane running kinematics.

HERS did not demonstrate any significant associations with running biomechanics. Together with the limited and inconsistent evidence in the literature [45, 46, 48], these findings do not support a connection between HERS and frontal-plane running biomechanics in this cohort. However, the contribution of HERS to running mechanics may depend on specific populations, tasks, or loading conditions and requires further investigation.

### Speed

Overall, the intervention did not produce significant changes in running biomechanics at any of the three tested speeds (Table 2). Therefore, no conclusions can be drawn regarding speed-dependent effects of the intervention. The absence of significant biomechanical changes across running speeds suggests that the implemented hip-strengthening program did not alter the evaluated aspects of running mechanics in healthy, well-trained runners.

In the cross-sectional analyses, the only significant finding was observed between greater HABDS and higher knee abduction moments during late stance at slow running speed. No significant associations were identified at medium or fast running speeds. However, because running speeds were analyzed separately and no formal interaction testing was performed, differences in the magnitude of strength–biomechanics relationships across speeds cannot be directly compared. Thus, the present findings do not provide evidence for consistent speed-related differences in the relationship between hip strength and frontal-plane running biomechanics. For the secondary biomechanical outcomes, no significant associations with HABDS were identified at any speed. Therefore, no conclusions regarding speed-dependent relationships for these outcomes can be drawn.

### Limitations

Some limitations warrant consideration. The statistical approach was shaped by SPM1D’s capabilities, which currently do not support mixed-effects models or repeated-measures ANCOVA with multiple factors and covariates. Consequently, running speeds were analyzed separately, and sex was included as a covariate in the cross-sectional analyses but addressed through stratified analyses in the intervention component. These approaches do not explicitly account for within-subject dependencies or higher-order interactions. Therefore, comparisons across speeds and sexes should be considered exploratory and interpreted descriptively rather than as formal tests of interaction effects. Nevertheless, this approach allowed us to analyze continuous time-series data across the entire stance phase within the SPM framework. This addresses an important limitation of previous studies that relied on discrete outcome variables (e.g., peak angles) and may have overlooked important features of the biomechanical waveform [24]. Accordingly, the findings regarding differences across speeds and sexes should not be interpreted as evidence of speed- or sex-dependent effects.

While HABDS gains were meaningful and comparable to previous studies [21, 22], improvements in HERS were modest. Although session adherence was relatively high, the absence of direct supervision may have limited training quality and thus attenuated overall strength development, as supervised resistance training typically produces greater strength and hypertrophy than unsupervised approaches [53, 54]. Moreover, self-reported adherence confirms completion of training sessions but does not verify exercise technique, range of motion, or training intensity, which may have affected intervention fidelity. However, this alone does not fully account for the comparatively small HERS gains. A more likely explanation is the specificity of training: strength gains tend to transfer best when training and testing closely match in movement and loading conditions [55]. Here, the HABDS test closely resembled the side-plank exercise, whereas the clamshell used to target HERS differed substantially from the dynamometer-based assessment. This mismatch may have limited transfer to the testing condition and contributed to the smaller observed HERS gains.

The hip strength-testing protocol was based on established approaches used in previous studies investigating hip strength and running biomechanics. Snyder et al. (2009), one of the early intervention studies in this field, assessed hip abductor strength in a side-lying position and hip external rotator strength in a seated position, using handheld dynamometers secured with straps [22]. Similar side-lying hip abductor testing has subsequently been used in several studies [26, 28, 43, 51, 56], while seated hip external rotator strength testing has also been reported [43, 48, 56]. Studies using stationary dynamometers have likewise assessed hip abductor strength in a side-lying position [46, 57] and hip external rotator strength in a seated position [45]. Sever et al. (2024) specifically used an ISOMED 2000 to assess hip abductor strength in a side-lying position [52]. In addition, previous reliability studies have demonstrated good-to-excellent reproducibility of strength measurements using the ISOMED 2000 across different muscle groups and testing modes [32, 34]. Nevertheless, no previous study has established the test–retest reliability of the exact hip strength testing protocol used in the present study, including the specific testing positions, stabilization, range of motion, contraction conditions, and the MVC-based testing procedure. Furthermore, familiarization was limited to the same testing session, and no separate familiarization session was conducted. These factors should therefore be considered when interpreting the reliability and sensitivity of the strength measurements.

In addition, the study population consisted of healthy, well-trained runners, which may limit the generalizability of the findings to recreational runners, individuals with lower training status, or runners with existing running-related injuries. This population characteristic should also be considered when interpreting the null biomechanical findings, as the relatively high training status and absence of injury may have limited the potential for further biomechanical adaptation. However, studying healthy runners allowed the effects of hip strengthening on running biomechanics to be examined without the potential confounding influence of current injury- or pain-related adaptations.

Although assessors were blinded during most assessments, the researcher conducting the post-intervention running analysis was aware of group allocation. Biomechanical processing was performed using pre-specified MATLAB scripts tested on pilot data, thereby reducing subjective processing decisions. However, assessor bias during post-intervention data processing cannot be excluded.

Finally, running intensity was not objectively monitored during the intervention period. Although participants were instructed to maintain their habitual running volume and intensity, only weekly running volume was objectively tracked using running watches. More comprehensive monitoring approaches (e.g., heart rate monitoring, session RPE, or training logs) could have provided additional information on training exposure. However, these approaches were considered less feasible within the study design. Similarly, habitual exposure to resistance training was not quantitatively monitored during the intervention. Participants were instructed to maintain their habitual strength training and not to initiate additional hip abductor or hip external rotator strengthening exercises. Nevertheless, differences in habitual resistance-training exposure between groups cannot be excluded and may have introduced additional variability in training exposure. Given the randomized controlled design, any uncontrolled variation in habitual training characteristics was expected to be distributed between groups.

### Practical implications and outlook

From a practical perspective, these findings should be interpreted specifically in the context of healthy, well-trained runners and the frontal-plane biomechanical outcomes examined in this study. Previous studies have reported reductions in pain and improvements in function following hip abductor and external rotator strengthening in symptomatic runners, particularly those with patellofemoral pain [58, 59]. In contrast, the present study involved healthy, well-trained runners with relatively high baseline function and strength levels. Notably, the 10-week intervention applied here was longer and of higher intensity and volume than previous studies [21, 22, 25–27], and likely exceeds what recreational runners typically perform independently. Therefore, in already well-trained and asymptomatic runners, expectations for meaningful changes in the frontal-plane running biomechanics examined here following isolated strength training alone should be modest.

Future research may clarify in which populations and under which training conditions hip strengthening translates into biomechanical adaptations, and whether combining it with neuromuscular or technique-based interventions yields more consistent changes in running mechanics.

## Conclusions

A 10-week isolated hip abductor and external rotator strengthening program resulted in moderate increases in HABDS and only modest improvements in HERS. However, these gains did not alter frontal-plane running biomechanics in this cohort of healthy, well-trained runners. These null biomechanical findings should be interpreted in the context of the participants’ high training status and the absence of injury. They may not generalize to less-trained or symptomatic populations. No consistent differences in intervention responses were observed across running speeds. Cross-sectionally, greater HABDS was associated with higher knee abduction moments during slow running, contrary to the primary hypothesis, whereas no significant associations were identified at medium or fast speeds. No other cross-sectional associations remained significant after correction for multiple comparisons. Although one association was observed under a specific running condition, the overall findings do not support a consistent relationship between hip strength and frontal-plane running biomechanics.

Overall, the primary finding of this study is that increases in hip strength following isolated strengthening did not result in measurable changes in frontal-plane running biomechanics. Future research should clarify in which populations and under which conditions hip strengthening leads to biomechanical adaptations, and whether combining it with neuromuscular or technique-based interventions yields more consistent effects.

## Supplementary Information

Supplementary Material 1.

Supplementary Material 2.

## Data Availability

The datasets used and/or analyzed during the current study are available from the corresponding author on reasonable request.
