# Supplementary material for: A 10-week randomized controlled hip-strengthening intervention in healthy, well-trained runners: longitudinal and cross-sectional analyses of frontal-plane running biomechanics across running speeds
Source: BMC Sports Sci Med Rehabil. 2026 Oct 6;18:470. doi: 10.1186/s13102-026-02148-z (PMC13647887; doi:10.1186/s13102-026-02148-z)
Supplement: Supplementary file 1 [file 13102_2026_2148_MOESM1_ESM.docx]

**CONSORT 2025 checklist of information to include when reporting a randomised trial***

| **Section / Topic** | **No** | **CONSORT 2025 checklist item description** | **Reported on page no.** |
| --- | --- | --- | --- |
| **Title and abstract** | | |  |
| Title and structured abstract | 1a | Identification as a randomised trial | Reported in title page |
|  | 1b | Structured summary of the trial design, methods, results, and conclusions | Reported in abstract |
| **Open science** | | |  |
| Trial registration | 2 | Name of trial registry, identifying number (with URL) and date of registration | This study was not prospectively registered. |
| Protocol and statistical analysis plan | 3 | Where the trial protocol and statistical analysis plan can be accessed | Reported in Methods section |
| Data sharing | 4 | Where and how the individual de-identified participant data (including data dictionary), statistical code and any other materials can be accessed | De-identified participant data and statistical code are available from the corresponding author upon reasonable request. |
| Funding and conflicts of interest | 5a | Sources of funding and other support (e.g., supply of drugs), and role of funders in the design, conduct, analysis and reporting of the trial | No external funding was received. |
|  | 5b | Financial and other conflicts of interest of the manuscript authors | Reported in Declarations section |
| **Introduction** | | |  |
| Background and rationale | 6 | Scientific background and rationale | Reported in Introduction |
| Objectives | 7 | Specific objectives related to benefits and harms | Reported in Introduction |
| **Methods** | | |  |
| Patient and public involvement | 8 | Details of patient or public involvement in the design, conduct and reporting of the trial | No patient or public involvement was included. |
| Trial design | 9 | Description of trial design including type of trial (e.g., parallel group, crossover), allocation ratio, and framework (e.g., superiority, equivalence, non-inferiority, exploratory) | Reported in Methods |
| Changes to trial protocol | 10 | Important changes to the trial after it commenced including any outcomes or analyses that were not prespecified, with reason | No important changes to the protocol were made after trial commencement. |
| Trial setting | 11 | Settings (e.g., community, hospital) and locations (e.g., countries, sites) where the trial was conducted | Reported in Methods |
| Eligibility criteria | 12a | Eligibility criteria for participants | Reported in Methods |
|  | 12b | If applicable, eligibility criteria for sites and for individuals delivering the interventions (e.g., surgeons, physiotherapists) | Not applicable. |
| Intervention and comparator | 13 | Intervention and comparator with sufficient details to allow replication. If relevant, where additional materials describing the intervention and comparator (e.g., intervention manual) can be accessed | Reported in Methods |
| Outcomes | 14 | Pre-specified primary and secondary outcomes, including the specific measurement variable (e.g., systolic blood pressure), analysis metric (e.g., change from baseline, final value, time to event), method of aggregation (e.g., median, proportion), and time point for each outcome | Reported in Methods |
| Harms | 15 | How harms were defined and assessed (e.g., systematically, non-systematically) | Exercise-related adverse events were monitored throughout the intervention; no serious adverse events occurred. |
| Sample size | 16a | How sample size was determined, including all assumptions supporting the sample size calculation | Reported in Methods |
|  | 16b | Explanation of any interim analyses and stopping guidelines | No interim analyses or stopping guidelines were planned. |
| Randomisation: |  |  |  |
| Sequence generation | 17a | Who generated the random allocation sequence and the method used | Reported in Methods |
|  | 17b | Type of randomisation and details of any restriction (e.g., stratification, blocking and block size) | Reported in Methods |
| Allocation concealment mechanism | 18 | Mechanism used to implement the random allocation sequence (e.g., central computer/telephone; sequentially numbered, opaque, sealed containers), describing any steps to conceal the sequence until interventions were assigned | Reported in Methods |
| Implementation | 19 | Whether the personnel who enrolled and those who assigned participants to the interventions had access to the random allocation sequence | Reported in Methods |
| Blinding | 20a | Who was blinded after assignment to interventions (e.g., participants, care providers, outcome assessors, data analysts) | Reported in Methods |
|  | 20b | If blinded, how blinding was achieved and description of the similarity of interventions | Reported in Methods |
| Statistical methods | 21a | Statistical methods used to compare groups for primary and secondary outcomes, including harms | Reported in Statistical analysis section |
|  | 21b | Definition of who is included in each analysis (e.g., all randomised participants), and in which group | Reported in Statistical analysis section |
|  | 21c | How missing data were handled in the analysis | No missing data were present for the 3D running analysis. Table 3 reports the sample size for each strength test variation. |
|  | 21d | Methods for any additional analyses (e.g., subgroup and sensitivity analyses), distinguishing prespecified from post-hoc | Reported in Statistical analysis section |
| **Results** | | |  |
| Participant flow, including flow diagram | 22a | For each group, the numbers of participants who were randomly assigned, received intended intervention, and were analysed for the primary outcome | Reported in Methods |
|  | 22b | For each group, losses and exclusions after randomisation, together with reasons | Reported in Methods |
| Recruitment | 23a | Dates defining the periods of recruitment and follow-up for outcomes of benefits and harms | Reported in Methods |
|  | 23b | If relevant, why the trial ended or was stopped | The trial was completed as planned. |
| Intervention and comparator delivery | 24a | Intervention and comparator as they were actually administered (e.g., where appropriate, who delivered the intervention/comparator, how participants adhered, whether they were delivered as intended [fidelity]) | Reported in Methods |
|  | 24b | Concomitant care received during the trial for each group | Reported in Methods |
| Baseline data | 25 | A table showing baseline demographic and clinical characteristics for each group | Reported in Table 1 |
| Numbers analysed,  outcomes and estimation | 26 | For each primary and secondary outcome, by group:   - the number of participants included in the analysis - the number of participants with available data at the outcome time point - result for each group, and the estimated effect size and its precision (such as 95% confidence interval) - for binary outcomes, presentation of both absolute and relative effect size | Reported in Table 2, 3, and 4; all 48 participants provided the data for Table 2 and 4. |
| Harms | 27 | All harms or unintended events in each group | No serious adverse events occurred during the intervention period. |
| Ancillary analyses | 28 | Any other analyses performed, including subgroup and sensitivity analyses, distinguishing pre-specified from post-hoc | Reported in Statistical analysis and Results sections |
| **Discussion** | | |  |
| Interpretation | 29 | Interpretation consistent with results, balancing benefits and harms, and considering other relevant evidence | Reported in Discussion |
| Limitations | 30 | Trial limitations, addressing sources of potential bias, imprecision, generalisability, and, if relevant, multiplicity of analyses | Reported in Discussion |

*We strongly recommend reading this statement in conjunction with the CONSORT 2025 Explanation and Elaboration and/or the CONSORT 2025 Expanded Checklist for important clarifications on all the items. We also recommend reading relevant CONSORT extensions. See [www.consort-spirit.org](http://www.consort-spirit.org).

Citation: Hopewell S, Chan AW, Collins GS, Hróbjartsson A, Moher D, Schulz KF, et al. CONSORT 2025 Statement: updated guideline for reporting randomised trials. BMJ. 2025; 388:e081123. <https://dx.doi.org/10.1136/bmj-2024-081123>.

© 2025 Hopewell et al. This is an Open Access article distributed under the terms of the Creative Commons Attribution License (<https://creativecommons.org/licenses/by/4.0/>), which permits unrestricted use, distribution, and reproduction in any medium, provided the original work is properly cited.
