# Supplementary material for: A 10-week randomized controlled hip-strengthening intervention in healthy, well-trained runners: longitudinal and cross-sectional analyses of frontal-plane running biomechanics across running speeds
Source: BMC Sports Sci Med Rehabil. 2026 Oct 6;18:470. doi: 10.1186/s13102-026-02148-z (PMC13647887; doi:10.1186/s13102-026-02148-z)
Supplement: Supplementary file 2 [file 13102_2026_2148_MOESM2_ESM.docx]

# Supplementary materials

**Supplementary Fig. 1.** Baseline frontal-plane right knee moments during stance for the intervention (blue) and control (grey) groups across running speeds (slow, medium, and fast). Solid lines represent the group mean and shaded areas indicate ±1 standard deviation. The figure is presented for descriptive comparison only. No statistical inference was performed.

**Supplementary Fig. 2.** Baseline frontal-plane right pelvis angles during stance for the intervention (blue) and control (grey) groups across running speeds (slow, medium, and fast). Solid lines represent the group mean and shaded areas indicate ±1 standard deviation. The figure is presented for descriptive comparison only. No statistical inference was performed.

**Supplementary Fig. 3.** Baseline frontal-plane right hip angles during stance for the intervention (blue) and control (grey) groups across running speeds (slow, medium, and fast). Solid lines represent the group mean and shaded areas indicate ±1 standard deviation. The figure is presented for descriptive comparison only. No statistical inference was performed.

**Supplementary Fig. 4.** Baseline frontal-plane right knee angles during stance for the intervention (blue) and control (grey) groups across running speeds (slow, medium, and fast). Solid lines represent the group mean and shaded areas indicate ±1 standard deviation. The figure is presented for descriptive comparison only. No statistical inference was performed.

**Supplementary Table 1** Absolute values and changes in hip abductor strength (HABDS) and hip external rotator strength (HERS) across groups, limbs, and contraction modes.

|  |  |  | **Pre (Nm/kg)** | | **Post (Nm/kg)** | | **Δ (Nm/kg) (95% CI)** | |
| --- | --- | --- | --- | --- | --- | --- | --- | --- |
| **Exercise** | **Side** | **Mode** | **Con** | **Int** | **Con** | **Int** | **Con** | **Int** |
| **HABDS** | **Left** | **iso** | 1.90 ± 0.36 | 1.95 ± 0.34 | 1.92 ± 0.28 | 2.15 ± 0.40 | +0.05  (-0.02 to +0.13) | +0.23  (+0.13 to +0.33) |
|  |  | **con** | 1.69 ± 0.32 | 1.70 ± 0.29 | 1.67 ± 0.25 | 1.87 ± 0.34 | +0.01  (-0.05 to +0.07) | +0.20  (+0.11 to +0.30) |
|  |  | **ecc** | 2.05 ± 0.39 | 2.07 ± 0.35 | 2.10 ± 0.29 | 2.36 ± 0.43 | +0.02  (-0.06 to +0.10) | +0.26  (+0.13 to +0.39) |
|  | **Right** | **iso** | 1.92 ± 0.33 | 1.90 ± 0.36 | 1.91 ± 0.34 | 2.11 ± 0.42 | -0.02  (-0.09 to +0.06) | +0.20  (+0.11 to +0.30) |
|  |  | **con** | 1.64 ± 0.28 | 1.59 ± 0.31 | 1.62 ± 0.32 | 1.83 ± 0.35 | -0.05  (-0.14 to +0.05) | +0.21  (+0.13 to +0.28) |
|  |  | **ecc** | 2.05 ± 0.37 | 2.03 ± 0.43 | 2.04 ± 0.41 | 2.33 ± 0.40 | -0.02  (-0.14 to +0.11) | +0.29  (+0.18 to +0.40) |
| **HERS** | **Left** | **iso** | 0.76 ± 0.13 | 0.75 ± 0.13 | 0.77 ± 0.18 | 0.81 ± 0.18 | -0.02  (-0.05 to +0.02) | +0.04  (+0.01 to +0.07) |
|  |  | **con** | 0.60 ± 0.12 | 0.58 ± 0.12 | 0.62 ± 0.15 | 0.64 ± 0.15 | -0.02  (-0.04 to +0.00) | +0.02  (-0.01 to +0.05) |
|  |  | **ecc** | 0.77 ± 0.14 | 0.74 ± 0.12 | 0.82 ± 0.16 | 0.87 ± 0.20 | -0.03  (-0.07 to +0.01) | +0.05  (+0.01 to +0.09) |
|  | **Right** | **iso** | 0.78 ± 0.15 | 0.78 ± 0.16 | 0.80 ± 0.19 | 0.81 ± 0.18 | -0.00  (-0.04 to +0.03) | +0.02  (-0.02 to +0.05) |
|  |  | **con** | 0.62 ± 0.12 | 0.60 ± 0.13 | 0.64 ± 0.15 | 0.65 ± 0.15 | -0.03  (-0.05 to +0.00) | +0.01  (-0.03 to +0.04) |
|  |  | **ecc** | 0.83 ± 0.14 | 0.79 ± 0.17 | 0.86 ± 0.16 | 0.90 ± 0.19 | -0.04  (-0.08 to +0.00) | +0.04  (+0.00 to +0.08) |
| Pre- and post-intervention values are presented as mean ± standard deviation (Nm/kg) for the control (Con) and intervention (Int) groups. Absolute changes (Δ Nm/kg) are presented as means with 95% confidence intervals and were calculated for each participant as post − pre, then averaged within groups. These descriptive within-group changes are not directly comparable to the adjusted between-group ANCOVA results reported in Table 3. | | | | | | | | |

**Supplementary Fig. 5** Distribution of absolute changes in body-mass-normalized [Nm/kg] hip abductor strength (HABDS) and hip external rotator strength (HERS) for control (grey) and intervention (blue) groups across contraction modes and limbs. Rows: contraction modes; columns: HABDS (left) and HERS (right). Boxes: interquartile range; central line: median; whiskers: 1.5× interquartile range; red crosses: outliers; open circles: individual participants; color-coded by sex (red = female, blue = male). Participants who are also outliers are displayed twice: once as an open circle and once as a cross.
